# From lab to life: how wearable devices can improve health equity

**DOI:** 10.1038/s41467-023-44634-9

**Published:** 2024-01-02

**Authors:** Jessica R. Walter, Shuai Xu, John A. Rogers

**Affiliations:** 1 Department of Obstetrics and Gynecology, Northwestern University, Chicago, IL 60611 USA; 2 Querrey Simpson Institute for Bioelectronics, Northwestern University, Evanston, IL 60611 USA; 3 Sibel Health, Chicago, IL 60614 USA; 4 Department of Biomedical Engineering, Northwestern University, Evanston, IL 60208 USA; 5 Department of Materials Science and Engineering, Northwestern University, Evanston, IL 60208 USA; 6 Department of Neurological Surgery, Northwestern University Feinberg School of Medicine, Chicago, IL 60611 USA

**Keywords:** Developing world, Translational research, Biosensors, Biomedical engineering

## Abstract

Wearable devices can provide personalised medicine at the point of need, potentially increasing access to health services and therefore improving health equity. Here the authors discuss their experiences developing wearable devices for vulnerable patient populations, including neonates and pregnant individuals.

## Teaser

Medical wearables, non-invasive devices that measure physiological biomarkers, are potentially disruptive and powerful tools to promote health equity at scale. Here we describe our experiences designing, validating, and deploying wearable sensors in vulnerable patient populations to improve health outcomes.

The Centers for Disease Control and Prevention defines health equity as the “state in which everyone has a fair and just opportunity to attain their highest level of health”^1^. Barriers to achieving health equity include poverty, institutionalized racism, environmental factors, and geography. Advances in technology expedited by the COVID-19 pandemic have increased interest in digital health, such as telemedicine, electronic health records, and artificial intelligence, as a tool to address health disparities.

Comparatively less focus has been given to a narrower, but fundamental component of the evolving digital health landscape—wearables. Medical wearables are noninvasively connected to the body and measure, store, and in some cases interpret biomarkers of clinical significance. Medical wearables are not confined to hospital settings, and ideally seamlessly coexist in clinical and non-clinical environments, remotely interfacing with the broader healthcare delivery ecosystem^2^. Early wearables gained traction as personal fitness devices, leveraging familiar form factors like watches, wristbands, or rings. The development of alternative, more discrete form factors (e.g., patches), variable mounting locations (e.g., chest), and additional biophysical and biochemical sensing capabilities (e.g., electrocardiogram, blood glucose levels), broadened potential applications in healthcare. Future innovations in this device class are expansive, including therapeutic drug or haptic delivery systems, miniaturization, transient technologies, population-level remote monitoring-enabled disease detection, and incorporation of artificial intelligence^3^.

**Fig. 1:**
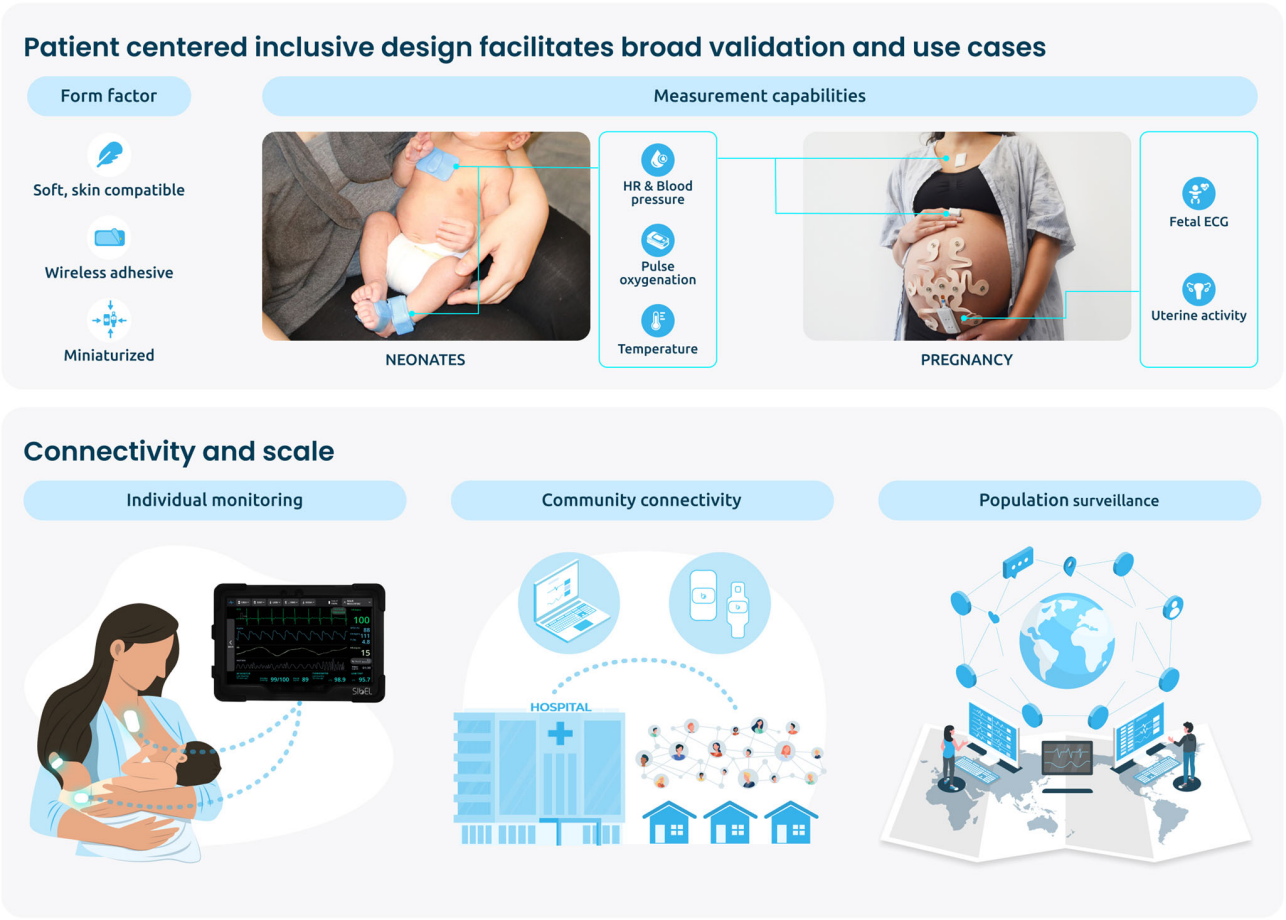
Application of advanced wearable devices for comprehensive monitoring of vulnerable patients at scale. Patient centered engineering and inclusive device design promote broad validation and various use cases. Deployment of low-profile sensors capable of comprehensive monitoring can encourage use in various vulnerable patient populations. Sensors can monitor an individual patient, producing physiological biomarkers for both personal and provider review in clinical and home environments. Low cost and reusable sensors facilitate scale.

Wearables, in cost-effective embodiments, are uniquely poised to promote health equity at scale, as these devices physically interact with a single patient, often in the lived environment, yet virtually connect this individual to families, caregivers, healthcare providers and their social and healthcare networks more generally. With intention and effort, the biomedical engineering community can steer the field of advanced wearables and flexible bioelectronics toward inclusive, accessible, and powerfully disruptive tools to promote health equity and reduce disparities. In this commentary we will share our experiences and those of others who are building, deploying, and scaling wearable sensors informed by the needs of arguably the most vulnerable patients—neonates and pregnant people—across a wide spectrum of resourced environments, to highlight wearable technology’s potential to improve clinical outcomes and promote health equity (Fig. 1).

Our team in the Querrey-Simpson Institute of Bioelectronics at Northwestern University, in partnerships with a startup company (Sibel Health), and corporate collaborators (Drager) developed a wireless wearable sensor system providing ICU-grade vital sign monitoring. The system was deployed in programs funded by the National Institutes of Health, the Bill and Melinda Gates Foundation, and Save the Children, to address the unmet challenge of caring for neonates, which is evidenced by a persistently highly mortality rate—with 1 million neonates dying on the first day of life, and 2 million more perishing within the first week of life globally^4^. Though outside the scope of this piece, it bears mentioning that neonatal outcomes vary profoundly, with a disproportionate burden of morbidity and mortality born by low- and middle-income countries (LMICs). Regardless of clinical setting, neonatal vital signs are traditionally collected with cumbersome, wired devices. Such devices are often adapted from those originally intended for adult use, and can result in iatrogenic injury, scarring, and disruption of the therapeutic skin-to-skin contact, known as Kangaroo Care, by a caregiver^5,6^. Advanced medical wearables, in soft, skin-like formats with capabilities in continuous wireless operation, are uniquely suited to serve their needs.

Limitations of current care informed the development of our system. Key innovations included a wireless, ultrathin, flexible, skin-like engineering design with a miniaturized physical footprint, compromising neither measurement accuracy nor comprehensiveness. In-sensor data analytics and real-time continuous data streaming provide access to pertinent biomarkers including heart rate, respiratory rate, pulse oxygenation, and skin temperature^7,8^. Furthermore, neonatal skin is fragile, requiring careful attention to mechanisms for skin adherence. We developed alternative adhesives and coupling mechanisms, leveraging weak Van der Waals forces to prioritize skin safety, while ensuring functionality and robust skin coupling. Advanced versions enhance skin tolerance through “holey architectures” increasing mechanical compliance and stretchability while also wicking moisture away from the skin-device interface, and reducing interface stresses and skin irritation with routine wear and removal^9^. Finally, engineering designs that prolong battery life, enable a reusable and wirelessly rechargeable mode of operation without external ports or receptacles, with low-cost consumables are critically important in scaling hardware solutions from the bench to hospitals, especially in challenging care environments.

Our early field testing on infants as young as 26 weeks (weighing <1500 g) relied upon local, well-resourced tertiary care facilities affiliated with Lurie Children’s Hospital and Northwestern Medicine Prentice Women’s Hospital. We additionally deployed devices for further validation and usability testing in Aga Khan University, Nairobi (AKU-N), a tertiary healthcare facility in Kenya^10^.

Others innovating in this emergent market include Neopenda, a public benefit corporation with extensive implementation work in Uganda and Kenya, who developed the neoGuard^TM^, a forehead-mounted device measuring neonatal vital signs. Laerdal Global Health is a Norwegian based not-for-profit sister company to Laerdal Medical, and markets (among other devices), the NeoBeat, a reusable and consumable-free device which is placed on the abdomen to measure newborn heart rate. Neobeat is functional on wet skin and can facilitate rapid neonatal resuscitation and risk stratification. In addition to general physiological monitoring, others have reported a specialized, low-cost wearable placed on the forehead to non-invasively detect jaundice and personalize phototherapy treatment through a transcutaneous bilirubinometer^11^.

In related efforts, we translated our experiences to another highly vulnerable health state—pregnancy. High rates of morbidity and mortality during pregnancy continue to occur in the United States and abroad, with more than 80% estimated as preventable^12^. Deepening health disparities underscore the urgency to revolutionize how we approach safety and monitoring of pregnant individuals to ensure equitable care and outcomes. Pregnancy indiscriminately occurs across the spectrum of the well to chronically ill, a broadening age range, and is further defined by progressive physiological changes superimposed on this baseline health. Antepartum and postpartum care occurs in a wide range of variably resourced environments (home, clinics, hospitals). Faced with increasing provider shortages that worsen geographically based inequities, remote patient monitoring with wearables has become increasingly relevant^13^. Finally, monitoring requires capabilities beyond routine vital signs, including measurements of uterine activity and fetal well-being (most often assessed by fetal heart rate, but also fetal electrocardiography and oximetry).

Principles of low profile, unobtrusive, yet comprehensive equipment for vital sign monitoring resonate with pregnant patients and staff. We expanded the sensor system to include an integrated monitoring platform using a wirelessly linked collection of three flexible electronic sensors to monitor the pregnant patient’s core vitals, uterine, and fetal activity. The sensors are time-synchronized via wireless connectivity and compatible with various low-cost mobile devices. The sensors additionally report advanced biomarkers including continuous cuffless blood pressure, electrohysterography-derived uterine monitoring, and automated body position classification. The system’s capacity to comprehensively monitor pregnant patients during labor has been validated via deployment in a large-scale longitudinal study in Zambia, India, and Ghana, of thousands of patients as part of the Limiting Adverse Birth Outcomes in Resource-Limited Settings (LABOR) study (NCT04102644)^14^. Future studies of this cohort are currently ongoing to assess if leveraging large volume datasets can produce population-specific predictive models designed to identify patients at risk of adverse outcomes, informing risk stratification and earlier intervention. InnAccel, an Indian-based company, is also investing in affordable and accessible pregnancy monitoring and has commercialized Fetal Lite, a single, wireless probe placed at the pregnant individual’s umbilicus that measures vital signs and fetal heart rate with remote connectivity and streaming capabilities, conveniently housed in an easily portable laptop-sized bag.

Advanced wearable technologies can be designed with capabilities well suited to overcome historical barriers to equitable global distribution and their meaningful use. It is estimated that more than 90% of medical devices used in LMICs are imported, having been neither designed, validated, nor produced locally. Sustainable use is further hampered by more than a third of these devices being rendered unusable by insufficient training, accessories, or incomplete supply chains^15^. However, wearables are intentionally designed to operate with equal effectiveness and accuracy in the clinic and home, with robust, ruggedized designs functional in various settings, including those often encountered in remote locations and/or LMICs (humidity, dust, extreme temperatures), in contrast to traditional medical devices used in sterile, controlled healthcare environments. In our experience, more than 5000 systems described herein have been deployed in 22 countries, used by 15,000 patients (including 500 neonates), aged 26 weeks to 98 years. Though tempting to focus efforts on scaling more simple devices, we hope our work and others demonstrate the highest levels of technological innovation belong and even thrive in low resource settings through intentional engineering and strategic partnerships. The clinical experiences of wearable technologies are still in the early stages, and a stepwise approach is warranted, defined by intentional and patient-informed engineering, rigorous validation of device accuracy, and pilot implementation studies. Ultimately, wearables should be expected to demonstrate a measurable improvement in health.

We note, however, the trajectory of medical wearables to promote health equity is not inevitable. To the contrary, the false assumption of device benevolence and data objectivity can perpetuate harm and generational injustices. For example, photoplethysmography (PPG)-derived heart rate and oxygen saturation, an affordable approach found in many devices, relies on green light signaling, which is notoriously inaccurate in patients with darker skin^16,17^. If measurement inaccuracies of wearable devices are disproportionately borne by historically marginalized populations, research, machine learning, or artificial intelligence built on these measures may perpetuate systemic racism and deepen health inequities, rather than uplift these communities. Another example, the continuous glucose monitor (CGM), is arguably one of few medical wearables with unequivocal demonstration of superior patient outcomes resulting from use, including improved glycemic control, quality of life, and morbidity among patients with Type 1 Diabetes with broad endorsements from guiding institutions such as the American Diabetes Association, and highlights disparities in access and use. Historically marginalized groups are less likely to use CGM at baseline or initiate use, even after controlling for patient age, disease, and socioeconomic factors^18^. Finally, generated data must be judiciously curated, interpreted, and evaluated to prevent reinforcing racial stereotypes or hierarchies. Consideration of population-specific comfort and amenability to use wearables due to concerns of data security and privacy, though outside the scope of this commentary, will be paramount to ensure devices and technologies at their core do not increase vulnerabilities. The technical infrastructure and digital platforms housing data derived from wearables cannot be an afterthought and should be built and regulated in parallel with their companion devices. Transparent information and formalized guidelines about best practices related to data ownership, storage, accessibility, and duration of data retention will all be essential to maintain user trust in wearables and algorithms derived from the data.

These challenges are not insurmountable; engineers, clinicians, scientists, and early adopters can actively work to promote tenets of equity and inclusion directly into the design process. Wearables can promote health equity at the individual patient level by prioritizing accurate and reproducible capture of physiology independent of the characteristics of the user, such as skin color, weight, or gender. Transparent validation testing in diverse patient populations, in both optimal laboratory conditions and real-world environments, will ensure accuracy of monitoring across the full range of patient characteristics^17,19^. In order to achieve meaningful impacts, technologies must obtain the relevant regulatory approvals (e.g., CE Mark or FDA clearance) and be designed specifically to enable scaled manufacturing at an affordable cost for all patients, independent of socioeconomic status. Formalized regulation of devices helps to ensure safe deployment and appropriate clinical application. Achievement of these clearances requires programmatic and institutional preparation, rigorous and transparent demonstration of system safety, device accuracy in alignment with industry standards, cybersecurity, and comprehensive risk assessments. Although labor intensive and expensive, these clearances are critical to maintaining patient and provider confidence, integrity of wearables and the data collected by them.

A vast majority of morbidity and mortality, particularly among the vulnerable patient populations highlighted in this perspective, is preventable with early detection and intervention. Wearables offer a unique opportunity to redefine what biomarkers we measure, how we capture this information, where data is collected, and the final frontier of how we make this physiological data actionable. Wearables are a unique part of the digital health revolution because they rely on a physical connection to a patient but can remotely connect that person to a vast healthcare infrastructure. Advanced wearables need not be overly complicated nor costly. To the contrary, prioritizing elegant, simple, and miniaturized designs through innovative engineering approaches will maximize their ability to promote health equity.

## Reporting summary

Further information on research design is available in the Nature Portfolio Reporting Summary linked to this article.

### Supplementary information

Reporting Summary
